# Maintained Smoking Cessation for 6 Months Equilibrates the Percentage of Sputum CD8^+^ Lymphocyte Cells with That of Nonsmokers

**DOI:** 10.1155/2009/812102

**Published:** 2010-02-23

**Authors:** Izolde Bouloukaki, Maria Tsoumakidou, Constantine I. Vardavas, Ioanna Mitrouska, Eleni Koutala, Nikolaos M. Siafakas, Sophia E. Schiza, Nikos Tzanakis

**Affiliations:** ^1^Department of Thoracic Medicine, University General Hospital, Medical School of the University of Crete, 71110 Heraklion, Greece; ^2^Department of Epidemiology, Social Medicine, Medical School of the University of Crete, 71110 Heraklion, Greece

## Abstract

Little is known about the longitudinal effects of smoking cessation on sputum inflammatory cells. We aimed to investigate the changes in sputum inflammatory cells and T-lymphocyte subpopulations after 6 and 12 months smoking cessation. Induced sputum was obtained from 68 healthy smokers before and after 6 months (*n* = 21) and 1 year (*n* = 14) smoking cessation and from ten healthy never-smokers. Inflammatory cells were identified by morphology and T-lymphocyte subpopulations by flow cytometry. Sputum macrophages were decreased after 12 months of smoking cessation in comparison to baseline, while neutrophils increased. Moreover, CD8^+^ T-cells were decreased in smokers before smoking cessation compared to never-smokers and increased in smokers after 6 months of smoking cessation in comparison to baseline; result that was maintained after 1 year of smoking cessation. These novel findings indicate that smoking cessation can equilibrate certain inflammatory cells of smokers with those of nonsmokers, within 6 months of smoking cessation.

## 1. Introduction

Smoking is the main risk factor for the development of a number of chronic lung diseases, such as chronic obstructive pulmonary disease (COPD) and lung cancer, and scientific evidence is conclusive of its role in the predisposition and outcome of several others [1]. While as stated above, active smoking is the main cause of lung cancer, smoking cessation significantly reduces the risk of lung cancer by 2-3 times at 10 years [2]. Moreover, sustained abstinence from smoking decreases the rate of decline in lung function to the levels of never-smokers and reduces the risk of developing clinically significant COPD [2]. These findings suggest that the adverse effects of smoking in the lungs are partly reversed after cessation. 

The mechanisms of cigarette smoke-induced lung injury have not been fully elucidated yet. However, it has been suggested that the early inflammatory responses to cigarette smoke are crucial to the development of subsequent tissue damage and disease in susceptible individuals. Furthermore, research has indicated that it may lead to a localised reaction and sequestration of neutrophils in pulmonary microvasculature and cause tissue damage through the release of toxic and chemotactic mediators, and thus further predispose to lung disease and inflammation [1–4]. Among lymphocytes, circulating CD4^+^ T-cell lymphocytes have been suggested as target cells for smoking. In particular memory CD4^+^ T-cell lymphocytes have been found to be selectively and dose dependently increased by active smoking, which is stimulated through the chronic procedure of habitual smoking [5]. In support of this, the number of inflammatory cells recovered by bronchoalveolar lavage fluid (BALF) is increased in smokers compared to never-smokers, primarily due to an increase in BALF macrophages [6–8]. Similarly to BALF, there is a significant increase in sputum and small airway macrophages in smokers [9–12]. Moreover, several reports have showed that neutrophils and lymphocytes are increased in smokers [6–9, 12–16]. CD4^+^T-cells in BALF of smokers have been reported to be decreased [15, 17] and CD8^+^ T-cells to be increased, resulting in a markedly decreased CD4^+^/CD8^+^ ratio compared to never-smokers [18, 19]. Similarly, in the small airway submucosa, there is a trend toward a decrease in CD4^+^ T-cells, an increase in CD8^+^ T-cells and a significant decrease in the CD4^+^/CD8^+^ T-cell ratio in smokers as compared to non-smokers [13]. 

Much less is known about the effects of smoking cessation. Two relatively old cross-sectional studies found no differences in inflammatory cells between smokers and exsmokers [20, 21], while others showed that BALF macrophage, but not neutrophil numbers are decreased in exsmokers compared to smokers [22]. In addition, it is worth noting that, in patients with COPD, one cross-sectional study in induced sputum found no differences in the cellular profiles and macrophage phenotypes when comparing smokers with ex-smokers [23], possibly indicating persistent inflammatory changes in the airways in patients who are predisposed to COPD. Longitudinal studies showed that BALF macrophages, neutrophils, lymphocytes and sputum macrophages decrease to the levels of never-smokers after smoking cessation [7, 24–26]. Reports on sputum neutrophils are controversial, showing either a decrease [25] or an increase [26]. There is only one longitudinal study in “healthy” smokers assessing T-lymphocyte subpopulations in bronchial biopsies and this study found no difference in CD8^+^ and CD4^+^ T-lymphocytes after smoking cessation [26]. No longitudinal studies have been reported the effect of smoking cessation on sputum T-lymphocyte subpopulations in “healthy” smokers. We have conducted a longitudinal study to examine whether changes in T lymphocyte subpopulations and airway inflammatory cells are reversed after a period of 6 months and 12 months smoking cessation in “healthy” smokers.

## 2. Materials and Methods

### 2.1. Subjects

In the University Hospital of Heraklion, Crete, induced sputum was obtained from 68 smokers recruited from the smoking cessation clinic, before their entrance into the smoking cessation protocol. All subjects provided written informed consent and ethical approval was provided by the University Hospitals Ethics Committee. Following enrollment, all smokers participated in the same behavioural therapy program and according to their degree of dependence (Fagerstrom test) they received nicotine replacement therapy and/or bupropion as part of their smoking cessation treatment. Ten never-smokers were recruited by advertisement and were the control subjects. All subjects were free of any respiratory tract infections at the time of the study or during the month preceding the study and at the follow-up at 6 and 12 months. Subjects did not suffer from any disease and did not receive any medication at the time of the first visit, while none had symptoms of chronic bronchitis. Furthermore, baseline lung functional tests were within the normal range and their chest radiograph was normal. The participating subjects' characteristics are depicted in Table 1.

### 2.2. Sputum Collection, Induction and Flow Cytometry

**Figure 1:**
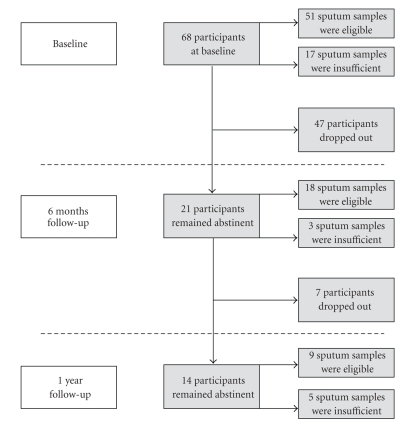
Flowchart of participation, smoking cessation and sputum quality.

Sputum specimens were successfully collected at baseline in all 10 controls and 68 smokers, while sufficient sputum of good quality was produced by 16 of the 21 successful quitters at baseline after 6 months of follow-up, in 18 out of 21 who had not relapsed (86% collection rate) and after 12 months of smoking cessation in 9 out of 14 quitters (65% collection rate). During each follow-up, confirmation of smoking status was assessed both by exhaled CO and urine cotinine levels. The progress of smoking cessation and sputum collection is depicted in Figure 1. 

 Sputum was induced and processed as previously described in [27]. Subjects inhaled hypertonic saline for three ten-minute sessions. The concentration of the inhaled saline was consecutively increased in each session from 3% to 4% and to 5%. Always between sessions the inhalation procedure was interrupted and the subjects were asked to blow their nose, rinse their mouth, and try to expectorate sputum into a sterilized box. By this way saliva contamination of the sample was minimized and the percentage of squamous cells in the sample was decreased. Sputum was processed within the next 30 minutes or no more than two hours, with the sample always kept in ice. The volume of the sputum was measured and sputum samples with a volume of at least 2 mL are reputed to be sufficient. The more viscid proportions of the sputum (plugs) were selected and weighed. Dithiothreitol (Calbiochem, Darmstadt, Germany) was added, followed by phosphate buffer saline (PBS). Then, the mixture was filtered and centrifuged. The cell pellet was resuspended with PBS, a total cell count of the sample was performed, and viability was tested by means of trypan blue exclusion method. If cell viability was less than 50% and/or squamous cell contamination more than 20%, the sample was not processed further. Sputum cytospin slides were stained using May-Grunwald Giemsa for differential cell count. Cell counting was performed by one investigator (IB), blind to the origin of the samples. At least 400 cells were counted. Cytospins with <50% of squamous cells and >400 nonsquamous cells were qualified as of good quality. Cell differential counts were expressed as % of total sputum nonsquamous cells.

**Figure 2:**
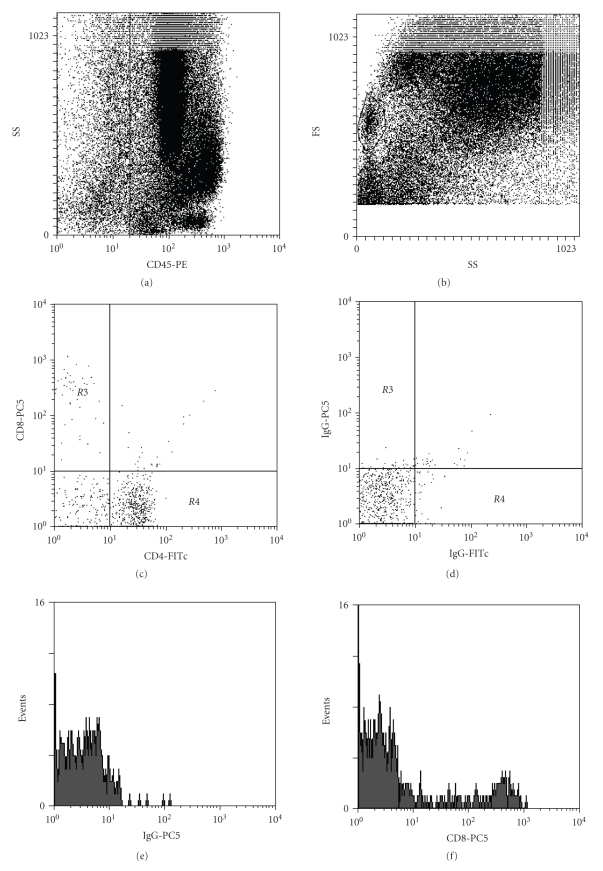

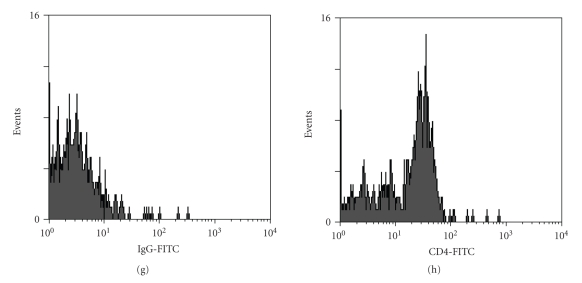
Example of a flow cytometric analysis with dot plots and histograms from a representative sample (from one subject). First, epithelial cells were excluded from the analysis by gating on all CD45^+^ Fleukocytes (plot (a)). Then lymphocytes were gated by morphology on an FS/SS plot (plot (b)). Finally, CD8^+^ and CD4^+^ lymphocytes were gated (plot (c)). Staining of isotype control antibodies is also shown (plot (d)). PE: phycoerythrin, PC-5: phycoerythrin-cyanine, FITC: fluorescein isothiocyanate.

Approximately 10^6^ sputum white cells were labelled for flow cytometry with monoclonal antibodies raised in mice against human (all from Immunotech; Marseille, France): phycoerythrin-cyanine (PECy-5) conjugated anti-CD8, fluorescein isothiocyanate (FITC) conjugated anti-CD4, and phycoerythrin (PE) conjugated anti-CD45 for 30 minutes at 4–8^*˚*^C or with isotype matched controls for 30 minutes at 4–8^*˚*^C. An ELITE COULTER cytometer was used. Data were acquired in list mode and analyzed using Epics Elite. The following gating strategy was applied: first dead cells, debris and epithelial cells were excluded on an SS-CD45 scatter plot, then lymphocytes were gated on an FS-SS scatter plot based on their size and granularity properties. Finally CD4 and CD8 positive cells were enumerated and expressed as percentage of % sputum lymphocytes on a CD4/CD8 scatter plot (Figure 2). Some samples were not sufficient enough (<10^6^ sputum white cells) to be labelled for flow cytometry and this accounts for the occasional missed cases in some cell counts (Figure 1).

### 2.3. Statistical Analysis

Normality was tested by the Shapiro-Wilk test. Variables were nonnormally distributed. In the group of subjects who successfully managed to quit smoking, differences in smokers before and after 6 and 12 months of smoking cessation were tested using the Friedman test followed by the Wilcoxon Signed Ranks test to see pairwise differences. Differences between smokers and the control group were tested using the Mann-Whitney *U* test. The software StatsDirect (Camcode*·* Cambridge, UK) was used. Probability values of <.05 were considered as statistically significant. 

## 3. Results

Sputum characteristics in smokers both before and after smoking cessation as also in the control population are presented in Table 2. As depicted the general characteristics of the samples (i.e., weight, total cell count, total cell count per gram, and sample viability) remained the same during the whole study, regardless of sampling time and subgroup. Moreover when comparing the samples of smokers at baseline with those of nonsmokers, the latter were found to have a higher percentage of sputum CD8^+^ lymphocyte subpopulations and subsequently a lower CD4^+^/CD8^+^ ratio (22.3 versus 13.9, *P* < .05 and 2.3 versus 3.8, *P* < .05, resp.). The percentages of inflammatory cells (neutrophils, macrophages) in the sputum was not found to differ between smokers and nonsmokers at baseline, and was not found to be altered in smokers after 6 months of smoking cessation. However, after 12 months of smoking cessation, the percentage of neutrophils was higher (61 versus 55, *P* < .05) and the percentage of macrophages was lower (34.5 versus 40.6, *P* < .05) when compared to baseline measurements. 

**Figure 3:**
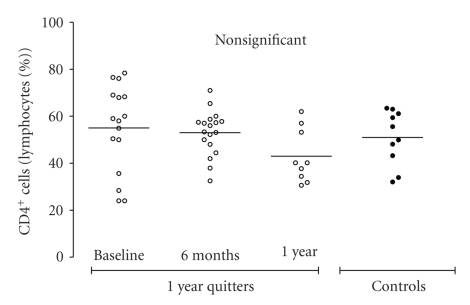
Percentage of sputum CD4^+^ T-cells (% sputum lymphocytes) in “healthy” smokers before smoking cessation (*n* = 16), after 6 months of smoking cessation (*n* = 18), and after 1 year of smoking cessation (*n* = 9). Results from 10 never-smokers (controls) are also presented.

**Figure 4:**
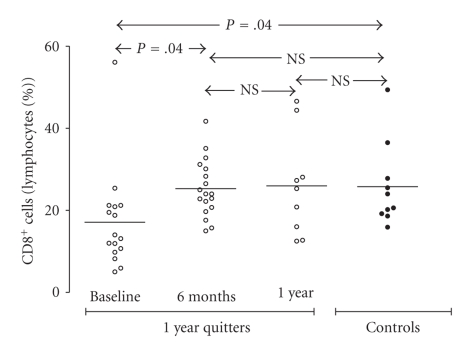
Percentage of sputum CD8^+^ T-cells (% sputum lymphocytes) in “healthy” smokers before smoking cessation (*n* = 16), after 6 months of smoking cessation (*n* = 18), and after 1 year of smoking cessation (*n* = 9). Results from 10 controls are also presented.

**Figure 5:**
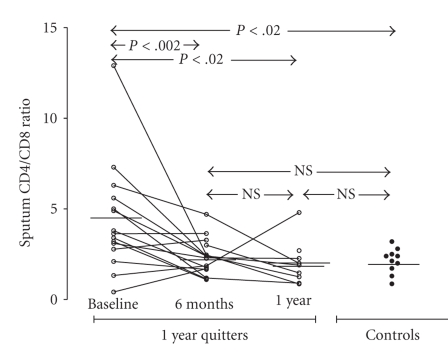
Percentage of sputum CD4^+^/CD8^+^ T-cells (% sputum lymphocytes) in “healthy” smokers before smoking cessation (*n* = 16), after 6 months of smoking cessation (*n* = 18), and after 1 year of smoking cessation (*n* = 9). Results from 10 controls are also presented.

Furthermore, as depicted in Figure 3, the percentage of CD4^+^ T-lymphocytes did not differ between smokers and controls. However, the number of CD4^+^ T-cells (expressed as a % of sputum lymphocytes) was not found to alter after cessation both after 6-month and 1-year follow-up. As noted above, a statistically significant lower percentage of CD8^+^ T-cells was observed in smokers in comparison to controls. Moreover, the percentage of CD8^+^ T-cells increased after 6 months of smoking cessation in quitters, and reached the controls' levels (Figure 4). During the months between month 6 and year 1 of follow-up, the percentage of CD8^+^ T-cells did not significantly differ. When the CD4^+^/CD8^+^ ratio was calculated, it was found to be more increased in smokers than in controls at baseline (Figure 5). Furthermore, it significantly decreased within 6 months of smoking, while as with the percentage of CD8^+^ cells, the CD4^+^/CD8^+^ ratio was not found to change after month 6 of cessation. 

## 4. Discussion

Our results indicated that smokers showed a lower percentage of CD8^+^ T-cells and an increase in the CD4^+^/CD8^+^ ratio compared to never-smokers at baseline. What is of interest though is that a significant increase in CD8^+^ T-lymphocytes and a decrease in the CD4^+^/CD8^+^ ratio were observed within 6 months of smoking cessation which reached the levels of controls. As, traditionally, the major activity of CD8^+^ T lymphocytes has been considered the facilitation of the rapid resolution of acute viral infections, the lower percentage of CD8^+^ T-cells suggests that smokers have a deficit in cell-mediated immunity in the lung, a site critical in the first-line defence against infection, and may explain the increased susceptibility of smokers for viral infections. Furthermore, previously our group has demonstrated that the expression of sputum dendritic cells maturation markers in smokers is decreased, which may significantly affect the ability of dendritic cells to induce effective immune responses, altering the lymphocyte subset population and therefore render smokers more susceptible to respiratory tract infections [28]. Moreover, after 12 months smoking cessation sputum macrophages decreased and neutrophils significantly increased among those who had quit. 

To the best of our knowledge there is very limited evidence that has assessed the alteration of T-lymphocyte subpopulations after smoking cessation. The present study is one of the first longitudinal studies that investigate the effect of smoking cessation on T-lymphocyte subpopulations in induced sputum of smokers with no concurrent disease or lung function impairment. 

Our findings seem to contradict the previously reported increase of CD8^+^ T-cells and the decrease in CD4^+^/CD8^+^ ratio in smokers compared with never-smokers in BALF and bronchial biopsies [13, 17–19]. Furthermore, a previous study on bronchial biopsies showed no difference in CD8^+^ and CD4^+^ T-lymphocytes before and after smoking cessation [26]. These discrepancies could reflect the different inflammatory microenvironment between airway lumen sampled by sputum and airway epithelium and mucosa sampled by bronchial biopsies [29]. Another possible explanation is that smoking suppresses the trans-epithelial migration of CD8^+^ lymphocytes, increasing their number in the large airway wall, while reducing their number in the airway lumen [30]. Therefore, it may be that in smokers, due to the abnormal trans-epithelial migration mechanism, CD8^+^ T-cells are increased in bronchial biopsies and decreased in the airway lumen, sampled by induced sputum. After smoking cessation, the trans-epithelial migration of CD8^+^ T-cells is restored and therefore higher numbers are detected in induced sputum [30]. 

The present study did not show any difference in sputum CD4^+^ T-cell numbers between smokers and never-smokers, as previously shown in [8, 16, 19, 26, 31]. Furthermore, no significant changes in the percentages of inflammatory cells (neutrophis, macrophages) in sputum were found in smokers compared to never-smokers. However, in agreement to the previous studies, the percentage of macrophages in sputum decreased after smoking cessation [7, 22, 24–27]. Additionally, the percentage of neutrophils significantly increased at 12 months smoking cessation, which is in agreement with a previous study in sputum [26]. It is possible that this noted increase in the percentages of sputum neutrophils could be due to increased apoptosis and transepithelial migration and may actually represent a protective mechanism to remove activated neutrophils from airway wall into the airway lumen [29]. However, the scientific evidence is inconclusive as other studies have shown that BALF neutrophils decrease to the levels of never-smokers at 6, 9 and 15 months after smoking cessation [7], while sputum neutrophils decrease at 12 months of smoking cessation [25]. 

The present study although novel and longitudinal, and therefore in a position to investigate into cause and effect did have some limitations that deserve comment. First, sputum samples cells primarily come from the lumen of the central airways and sputum changes in inflammatory cells and T-cells do not necessarily reflect changes in airway wall. However, common methods to study tissue, such as the examination of bronchial biopsies or surgical specimens cannot be easily applied on longitudinal studies. Second, as sputum has to be homogenized before flow cytometry, a possible effect of dithiothreitol (DTT) treatment on lymphocyte antigens has to be considered. The reducing agent DTT could have altered the CD8 antigen and interfered with the recognition of the antibody. In the present study a detailed analysis of the effects of DTT on the appropriate surface markers was not performed. However, Loppow et al. investigated peripheral blood leukocytes as a model for induced sputum cells and showed that the fluorescence intensity of CD8 was not altered by DTT, while there was statistically significant (*P* < .001), although small, changes in the percentages of CD8 positive lymphocytes. The authors concluded that comparability between samples concerning lymphocyte surface markers is preserved, suggesting that treatment of sputum samples with dithiothreitol does not invalidate the immunocytochemical analysis of lymphocytes [32]. Moreover, the mean age in the smokers' group was greater than in the control group and it could be argued that differences in lymphocyte subpopulations between smokers and never-smokers are due to age difference. However, it has been shown that age predominately reduces the numbers of CD4^+^ T-cells, while we found only a difference in CD8^+^ T-cells [33, 34]. Moreover, all our subjects were below 65 years and changes in T-cells have been mostly reported in subjects above that age. Furthermore, it is possible that gender factors could play a role, as the data presented were mainly based on that of male subjects. Another limitation of the study was the relatively small number of subjects, especially successful quitters after one year of smoking cessation. This is due to the increased relapse rate of smoking within 1 year. Taking the above into account, further studies, using a larger number of smokers, are needed to investigate the exact mechanism.

## 5. Conclusions

Conclusively, our results indicate that smoking cessation increases the percentage of CD8^+^ T-cells and decreases the CD4^+^/CD8^+^ ratio within 6 months of smoking cessation to that of the level of never-smokers, and is retained at that level among those abstinent at 12 months. These results indicate that some aspects of inflammation and immunological processes taking place in the airways of smokers with no existing lung impairment or concurrent respiratory disease can be reversed in those subjects who successfully quit smoking. 

## Figures and Tables

**Table 1:** Subject characteristics.

	Smokers			Nonsmokers (controls)	*P*-value*
	Total	Successful quitters at 6 months	Nonquitters		
**N**	68	21	47	10	N/A
Sex, M/F	59/9	19/2	40/7	8/2	*P* = .6
Age, (in years)	49 ± 8	49 ± 7	49 ± 9	33 ± 9	*P* = .002
Smoking (pack-years)	57 ± 18	53 ± 15	59 ± 19	N/A	N/A
FEV1, %pred Postbronchod.	105 ± 12	109 ± 13	104 ± 11	116 ± 13	*P* = .2
ΔFEV1 % pred	2.1 ± 1.8	2.4 ± 2.3	1.9 ± 2.2	1.9 ± 1.4	*P* = *.16 *
Fagerstrom Score	8 ± 1	7 ± 1	8 ± 1	N/A	N/A

Data are presented as mean ± SD, or in absolute numbers, N/A: non applicable, FEV1: forced expiratory volume in one second; ΔFEV1: change in FEV1 after bronchodilation.

*P-value between controls and successful quitters.

**Table 2:** Sputum differential cell counts and lymphocyte subpopulations.

		Smokers			Controls
	Total baseline	Successful quitters	Successful quitters	Successful quitters	(*n* = 10)
	(*n* = 51)	baseline	6 months	12 months	
		(*n* = 16)	(*n* = 18)	(*n* = 9)	
Weight, gr	1.2 (0.08–4.3)	1.4 (0.3–3.5)	0.89 (0.05–3.45)	1.08 (0.31–3.18)	1.41 (0.35–5.04)
TCC, ×10^6^ cells	2.78 (0.06–13.2)	2.5 (0.1–11.2)	4.02 (0.59–11.8)	3.85 (0.47–19.6)	5.29 (1.16–7.00)
TCC/gr	2.27 (0.03–13.4)	1.6 (0.03–11.6)	4.78 (0.49–11.8)	2.86 (0.51–46.67)	2.18 (0.68–6.71)
Viability %	80.42 (50–98.6)	80.4 (50.6–96.9)	92.62 (50–99.37)	89.16 (70.37–98.53)	84.71 (67.01–98.79)
SQUAM	10 (0.01–49.9)	10.6 (0.01–43.7)	6.4 (0–20)	5 (0.49–39)	10.78 (2.31–48.5)
NEUTR %	55.6 (3.3–87.3)	48.8 (17.7–80)	55.3 (17–88)	61 (3.6–84.8)*	40.5 (3.6–94)
MACRO %	40.6 (12.3–94)	49.1 (20–80.3)	41.8 (11.2–82.2)	34.5 (14.5–57.3)	55 (5.4–94)
LYMPHO %	0.12 (0–6.2)	0.1 (0–6.2.7)	0.25 (0–1.5)	0.5 (0–1.6)	0.55 (0–4.1)
EOS %	0 (0–3.6)	0 (0–0)	0 (0–0)	0 (0–7)	0 (0–0.5)
CD4^+^ %	59 (20–84)	58.5 (24–78.4)	54.7 (32.5–71)	40.2 (30.6–62)	53 (32–63)
CD8^+^ %	13.9 (4–56)	13.6 (5–56)	24 (15–42)*	25.3 (12.5–46.6)*	**22.3 (16–49)** ^†^
CD4^+^/CD8^+^ ratio	3.8 (0.4–16.2)	3.7 (0.4–12.9)	2.3 (1.1–4.7)*	1.9 (0.9–4.8)*	**2.3 (0.9–3.2)** ^†^

TCC: total cell count, SQUAM: squamous cells, NEUTR: neutrophils, MACRO: macrophages, LYMPHO: lymphocytes, EOS: eosinophils.

Data are presented as median (range). Squamous cells are expressed as the percentage of all cells, differential cell counts are presented % of total sputum nonsquamous cells, lymphocyte subpopulations are presented % of total sputum lymphocytes.

*P < .05 versus successful quitters at baseline, ^†^ *P* < .05 between controls and successful quitters at baseline.
